# Polyphyly in widespread *Salmonella enterica* serovars and using genomic proximity to choose the best reference genome for bioinformatics analyses

**DOI:** 10.3389/fpubh.2022.963188

**Published:** 2022-09-08

**Authors:** Emeline Cherchame, Guy Ilango, Véronique Noël, Sabrina Cadel-Six

**Affiliations:** Anses, Laboratory for Food Safety, Salmonella and Listeria Unit, Maisons-Alfort, France

**Keywords:** *Salmonella enterica*, genomic proximity, polyphyletic serovars, MLST profile, cgMLST, pan-phylogenetic analysis, reference complete genomes

## Abstract

*Salmonella* is the most common cause of gastroenteritis in the world. Over the past 5 years, whole-genome analysis has led to the high-resolution characterization of clinical and foodborne *Salmonella* responsible for typhoid fever, foodborne illness or contamination of the agro-food chain. Whole-genome analyses are simplified by the availability of high-quality, complete genomes for mapping analysis and for calculating the pairwise distance between genomes, but unfortunately some difficulties may still remain. For some serovars, the complete genome is not available, or some serovars are polyphyletic and knowing the serovar alone is not sufficient for choosing the most appropriate reference genome. For these serovars, it is essential to identify the genetically closest complete genome to be able to carry out precise genome analyses. In this study, we explored the genomic proximity of 650 genomes of the 58 *Salmonella enterica* subsp. *enterica* serovars most frequently isolated in humans and from the food chain in the United States (US) and in Europe (EU), with a special focus on France. For each serovar, to take into account their genomic diversity, we included all the multilocus sequence type (MLST) profiles represented in EnteroBase with 10 or more genomes (on 19 July 2021). A phylogenetic analysis using both core- and pan-genome approaches was carried out to identify the genomic proximity of all the *Salmonella* studied and 20 polyphyletic serovars that have not yet been described in the literature. This study determined the genetic proximity between all 58 serovars studied and revealed polyphyletic serovars, their genomic lineages and MLST profiles. Finally, we enhanced the open-access databases with 73 new genomes and produced a list of high-quality complete reference genomes for 48 *S. enterica* subsp. *enterica* serovars among the most isolated in the US, EU, and France.

## Introduction

For routine disease surveillance activities and outbreak investigations, the use of whole-genome sequencing (WGS) to identify and subtype foodborne bacterial pathogens has replaced traditional slide agglutination methods; likewise, to cluster and associate epidemiological strains, core genome multilocus sequence type (cgMLST) and single nucleotide polymorphism (SNP) analyses have replaced pulsed-field gel electrophoresis (PFGE) and multiple loci VNTR (MLVA) analyses. To meet the needs of real-time surveillance and ensure public health and economic benefits, the analysis of the complete genome is now routine for many reference laboratories around the world. cgMLST and SNP analyses are fast and several user-friendly tools exist for investigations of outbreak clusters (1–4). Nevertheless, although SNP phylogenetic core-genome analysis enables more detailed clustering between strains and better calculation of genomic distances between genomes, it requires complete genomes for processing the obtained data (3, 5). To ensure good-quality, complete reference genomes, which is essential for these epidemiological association analyses, we recently developed an open-source tool (SalmoDEST) that can download well-characterized good-quality, complete reference genomes from the open-access GenBank database (6). This tool can extract complete *Salmonella* genomes with a coverage higher than 50x and genome length over 4 Mb; it verifies the serovar to which genome belongs and identifies the corresponding MLST profile (6).

Nevertheless, although the number of compete genomes deposited in the open-access databases increases every year, a complete reference genome is still not available for several *Salmonella* serovars. The choice of a good reference genome is critical to ensure the sensitivity of the analyses performed when analyzing closely related genomes (5, 7). Selecting a reference genome close to the strains under study increases the fraction of the genome on which SNP variants can be screened for, thereby increasing method sensitivity. For instance, we have shown that the use of the reference genome Typhimurium LT2 led to an 11% loss of core genome information (89% of breadth coverage) in the SNP phylogenetic investigation of the *Salmonella* Wellikade outbreak occurred in 2016 in France (5). However, choosing the *S*. Gaminara strain SA20063285 reference genome provided 92% breadth coverage, corresponding to a loss of only 8% of core-genome information (5). When the complete genome is not available for the serovar studied, we proposed an operating protocol (5) that can be used in any laboratory involved in surveillance activities, outbreak management and emergency preparedness (5). The protocol identifies the closest complete genome to use for SNP phylogenetic analysis among the ones available in the EnteroBase *Salmonella* database (1, 8). We indicate how to query EnteroBase by searching for the closest hierarchical cluster (HC) 2,000 profile of the serovar under study and visualize results using the GrapeTree clustering analysis (5, 9).

Finally, when choosing the most suitable complete genome, polyphyletic serovars require special attention. A polyphyletic serovar derives from multiple independent ancestors (1). For example, a study of the phylogeny of the *Salmonella* Derby serovar showed that strains displaying the same antigenic pattern *S*. 1,4,[5],12: f,g: (10, 11) according to the White-Kauffmann-Le Minor scheme (12) — and, consequently, sharing the name *Salmonella* Derby — belonged to at least three distinct genomic lineages (13). A similar situation was reported for *Salmonella* Newport in 2013 (14). For *Salmonella* Derby, the three lineages were fully consistent with thoses identified by MLST analysis and were named according to their ST profile names (ST40, ST71, and ST682). The strains belonging to the ST40 lineage were distinct from those belonging to the ST71 lineage, differing by 26,957 SNPs with a standard deviation (SD) of 1,583. The genomes belonging to the ST682 lineage were the most genetically distant from ST40 and ST71, with an average of 33,961 SNPs and an SD of 4,102 SNPs (13). With such genomic distances between lineages, it seems evident that the choice of the appropriate reference genome for polyphyletic serovars is critical and cannot be based only on serovar name.

With the goal of providing a ready-to-use map of the genomic diversity of the *Salmonella enterica* subsp. *enterica* serovars prevalent in human health, animal health and the food sector, we carried out a phylogenetic study of the most frequently isolated serovars to give an overview of the main polyphyletic serovars and their genomic lineages.

## Materials and methods

### Selection of serovars and genomes

The serovars analyzed in this study are those identified as being the most frequently isolated in humans and the agri-food chain over a period of 10 years (from 2006 to 2016) in the United States (US), Europe (EU) and France (FR). The list of the most frequently isolated serovars was compiled based on data reported by the CDC, the USDA, the ECDC and the EFSA reports (11, 15–18). For FR, data from the official controls collected by the *Salmonella* Network, part of the Anses Laboratory for Food Safety (LSAl), and reports from the National *Salmonella* Reference Center were taken into account (10, 19). More than 1.5 million reported human cases and, animal and food isolates were compiled in six lists according to serovar prevalence. Three lists (i.e., one list for the US, one for EU and one for FR) were compiled for the serovars isolated from humans and three other lists for those collected from the agri-food sector. The three lists for human cases and the three lists for the agri-food isolates were used separately for the Venn diagram analysis that was carried out using the ggVennDiagram R package (v.1.2.1) (20). Finally, the serovars selected for this study were chosen according to the following criteria: being common to at least two lists and belonging to the leading 20 serovars of each list (Table 1).

**Table 1:** List of the 58 *Salmonella* serovars identified as being the most frequently isolated in humans and the agri-food sector over a period of 10 years (from 2006 to 2016) in the United States, Europe and France.

Agona	Derby	Johannesburg^b^	Muenster	Schwarzengrund
Albany	Dublin	Kedougou^c^	Napoli	Senftenberg
Anatum	Enteritidis	Kentucky	Newport	Stanley^c^
Banana^a^	Gallinarum^c^	Kottbus	Ohio	Tennessee
Bareilly^b^	Give	Livingstone	Oranienburg	Thompson
Bovismorbificans	Goldcoast^c^	London	Panama	Typhi
Braenderup	Hadar	Manhattan	Paratyphi B and Java	Typhimurium
Brandenburg	Havana	Mbandaka	Poona	Uganda
Bredeney	Heidelberg	Minnesota	Reading^b^	Veneziana^a^
Cerro	Indiana	Mississippi^b^	Rissen	Virchow
Chester	Infantis	Montevideo	*S*. 1,4,[5],12:i:-	
Coeln	Javiana^b^	Muenchen	Saintpaul	

**Figure 1:**
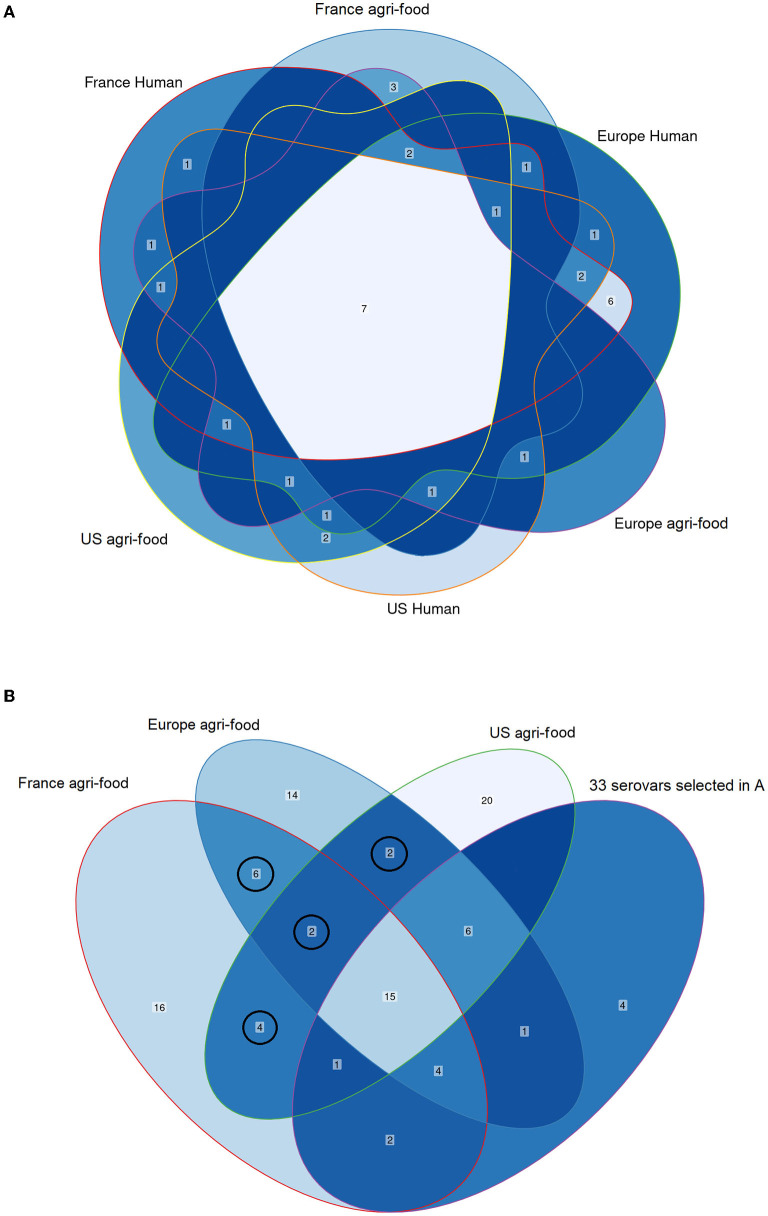
Venn diagrams illustrating the intersection between the most frequently isolated serovars in the United States (US), Europe (EU) and France (FR) from human cases and from the agri-food sector. Venn analysis was carried out in two steps showed in **(A,B)**. **(A)** Intersections between the top 25 serovars in human cases and the agri-food sector. The leading 25 human US, EU and FR serovars are included in the orange-, green- and red-outlined areas, respectively. The leading 25 agri-food sector US, EU and FR serovars are included in the yellow-, purple- and blue-outlined areas, respectively. The logical relation between the top 25 serovars in human cases and agri-food sector revealed 33 common serovars. The numbers within the intersections correspond to the common serovars. **(B)** Intersections between the leading 50 US, EU and FR agri-food sector serovars with the 33 serovars previously selected in **(A)**. The top 50 US, EU and FR agri-food sector serovars are included in the green-, blue- and red-shaded areas, respectively. The previous 33 selected serovars in **(A)** are included in the purple-shaded area. The logical relation between these four groups revealed 14 other common serovars. The new 14 common serovars are surrounded by black circles. The numbers within the intersections correspond to common serovars. The resulting 47 common serovars obtained by Venn analyses are showed in the Supplementary Figure 1.

Forty-seven serovars were selected because they were common to the United States, Europe and France according to the Venn analyses in Figure 1. Eleven other serovars (visualized by gray fill color in the list) were added because they belong to the top 20 serovars from each country but were not common to all countries. a: serovar from to the top 20 in FR; b: serovars from the top 20 in the US; c: serovars from the top 20 in EU.

For each of the serovars selected, the most common MLST profiles were identified using the data available in the EnteroBase *Salmonella* database on 19 July 2021. The MLST profiles with 10 or more genomes in the EnteroBase database were selected for this study. For each of these MLST profiles, three good-quality genomes were downloaded. The complete or contig genomes were searched and downloaded using the SalmoDEST tool (6) and manually *via* the GenBank and EnteroBase *Salmonella* databases. Good-quality genome criteria were a length > 4 Mb, coverage > 50x and an analysis of how well genome matched the predicted serovar using SeqSero2 (21). When available, genomes from the Anses LSAl collection were selected and sequenced for this study. One genome of *S*. Javiana was obtained from the strain S11LNR1976 (renamed 2019LSAL01686) from the French National Reference Laboratory Collection (LNR-Anses) in Ploufragan-Plouzané-Niort Laboratory. Three genomes of *S*. Paratyphi B were obtained from the strains CIP 106179, CIP 55.42 and CIP 106950 (renamed 2019LSAL01933, 2019LSAL01934 and 2019LSAL01936, respectively) of the French CIP collection (*Collection de l'Institut Pasteut*, Paris, https://www.pasteur.fr/en/public-health/biobanks-and-collections/collection-institut-pasteur-cip).

### Whole-genome sequencing analyses

#### Sequencing and assembly

Seventy-three genomes from Anses *Salmonella* Network collection were sequenced using the Illumina system producing paired-end reads as described in Cadel-Six et al. (22). The quality control, normalization and assembly were carried out with an in-house workflow called ARtWORK (23). The serovar and the multilocus sequence type (MLST) were attributed using the SeqSero2 (21) and MLSTseeman tools (24).

#### cgMLST analysis

The core-genome MLST (cgMLST) analysis was carried out with SeqSphere+ (Ridom® GmbH, Münster, Germany) under the EnteroBase cgMLST scheme based on 3002 loci (25).

#### Pan-genome phylogenetic analysis

The pan-genome kmer phylogenetic analysis was carried out with the QuickPhylo workflow as previously described (26), setting the Mash tool parameter to 1,000 selected kmers of 15 bases (27) and setting the DendroPy tool parameter to the neighbor-joining (NJ) method (28).

#### Tree annotation

Trees were visualized and annotated using R with the ggtree package (20, 29, 30).

## Results

### *Salmonella* serovars and genome selection

Fifty-eight *S. enterica* subsp. *enterica* serovars, the most frequently isolated in human cases and the agri-food sector in the US, EU and FR were selected for this study. The Venn analyses allowed selecting 47 prevalent common serovars in the US, EU and FR. The Venn diagrams in Figure 1 illustrate the intersections between the 25 and 50 most isolated serovars in humans and the agri-food sector in the US, EU and FR (Figure 1). The distribution of these 47 common serovars is illustrated in Supplementary Figure 1. Eleven other serovars were added because they belong to the leading 20 serovars of each list and were absent from the previous list comprising 47 common serovars. The 58 final serovars retained for this study are showed in Table 1.

For these 58 major *S. enterica* subsp. *enterica* serovars, 639 genomes were collected from GeneBank, EnteroBase, CIP and the Anses *Salmonella* Network databases following the criteria described above. Eleven *Salmonella* samples from the other subspecies were also included. Genome ID, accession number, predicted serovar, MLST profile, genome length and coverage of the 650 genomes retained in this study is reported in Supplementary Table 1. Of the final total set of 650 genomes, 83 were complete genomes and 567 were contigs. Among the complete genomes, 77 belonged to the *S. enterica* subsp. *enterica* and 6 to other subspecies. Of the 567 contig genomes selected, 562 belonged to the subspecies *enterica* and 5 contig genomes belonged to other subspecies (Supplementary Table 1).

### Phylogenetic analyses

The total set of 650 genomes was used for the first cgMLST analysis. From this analysis, a subset of 219 genomes were selected for the final phylogenetic cgMLST and pan-genome analyses. To conserve the genomic diversity of the first panel of 650 genomes, one genome per MLST profile was selected, favoring complete genomes when available. The subset of 219 genomes was composed of 74 complete genomes and 145 genomes in contigs. Genomes selected for the second 219 genome subset are shown in bold and gray fill color in the Supplementary Table 1.

#### cgMLST analysis of the panel of 650 genomes

**Figure 2:**
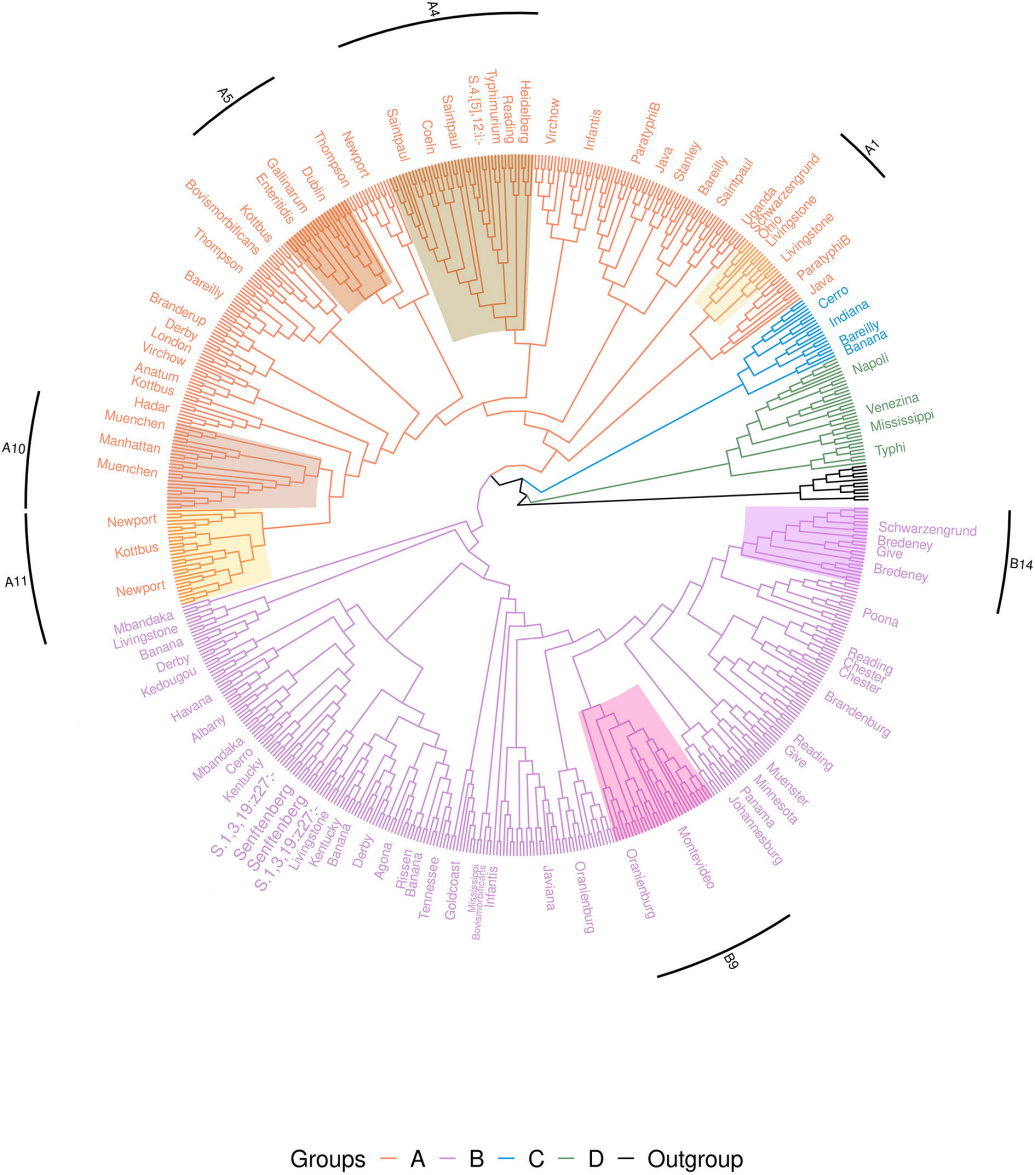
Phylogenetic cgMLST distance tree of the 650 genome set of *Salmonella*. The tree is rooted on the *Salmonella entrerica* subsp. *arizonae, diarizonae, houtenae, indica* and *salamae* genomes. For better visualization of the groups, the tree is shown without branch lengths. Branches are colored to distinguish the four groups. Due to the high number of strains in the tree, strain labels are not shown and serovars are indicated along corresponding branches. Strongly supported subgroups are shaded in different colors (for the description of these subgroups see also Figure 3 and **Table 3**).

Among the 650 genomes, the 11 genomes belonging to the other subspecies were used as outgroups. The cgMLST analysis shows that the 639 genomes belonging to *S. enterica* subsp. *enterica* were separated into four groups. Two of these groups, called groups A and B, included 90% of genomes (*n* = 580/639) and 50 of the 58 serovars studied. The 10% of the remaining *S. enterica* subsp. *enterica* genomes clustered into two separate groups, groups C and D (Figure 2).

Group A included all genomes of serovars Anatum, Braenderup, Coeln, Dublin, Enteritidis, Gallinarum, Hadar, Heidelberg, Java, Kottbus, London, Manhattan, Muenchen, Newport, Ohio, Paratyphi B, Saintpaul, Stanley, Thompson, Typhimurium, 4,[5],12:i:-, Uganda and Virchow. Group A also included the genomes belonging to the MLST profiles Bareilly ST203, 362, 464, 909, 1,612, 2,129, 2,270, 2,553, Bovismorbificans ST142, 377, 1,499, Derby ST682, Infantis ST32, 603, 2,283, 2,146, Livingstone ST543, 1,941, 2,247, Reading ST1628 and Schwarzengrund ST2250.

Group B included all genomes of the serovars Agona, Albany, Brandenburg, Bredeney, Chester, Give, Goldcoast, Havana, Javiana, Johannesburg, Kedougou, Kentucky, Mbandaka, Minnesota, Montevideo, Muenster, Oranienburg, Panama, Poona, Rissen, Senftenberg, 1,3,19:z27:- and Tennessee. Along with these last genomes, the Group B included also the genomes belonging to the MLST profiles Banana ST683, 1,035, 4,745, 5,220, Bovismorbificans ST50, Cerro ST1291, Derby ST39, 40, 71, 72, Infantis ST79, Livingstone ST457, 638, Mississippi ST425, Reading ST93, 412 and Schwarzengrund ST96, 322.

Group C included all genomes of serovar Indiana, the genomes of serovar Cerro characterized by the MLST profiles ST367, 1,593, 2,407, Banana ST7024 and Bareilly ST5146.

Finally, Group D included all genomes of serovars Typhi, Veneziana, Napoli and the genomes of the serovar Mississippi characterized by MLST profiles ST448 and 5,834 (Figure 2 and Supplementary Figure 2).

#### cgMLST and pan-phylogenetic analyses of the subpanel of 219 genomes

**Figure 3:**
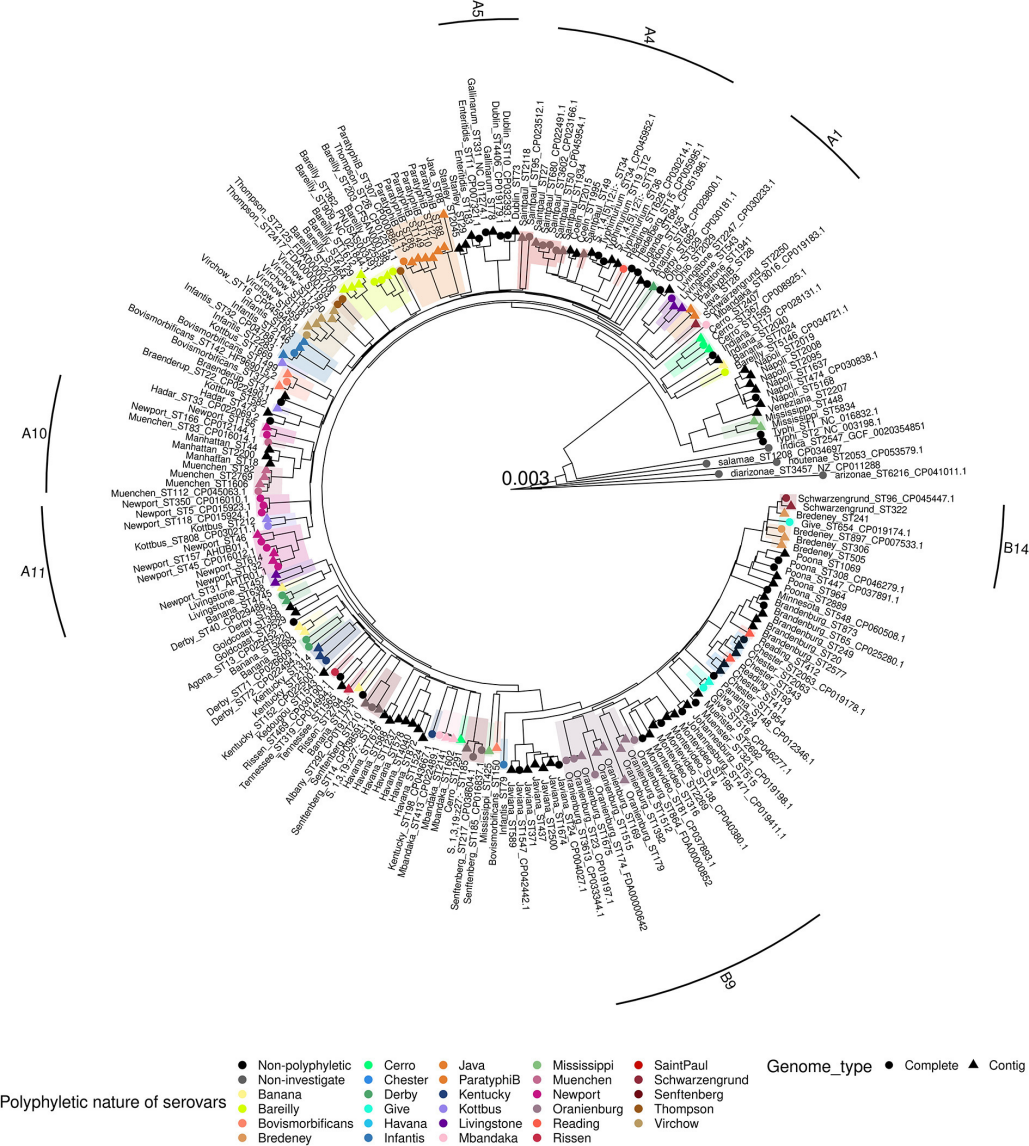
Phylogenetic pan-genome kmer distance tree of the 219 genome subset of *Salmonella*. The tree is rooted on the *Salmonella entrerica* subsp. *arizonae, diarizonae, houtenae, indica* and *salamae* genomes. The tree is shown with branch lengths. Polyphyletic serovars are shaded in different colors. Complete genomes are indicated with circles and contigs with triangles. Labels contain the serovar and the MLST profile of each strain. The strongly supported subgroups listed in the **Table 3** are highlighted in the margin of the tree. For example, A1 corresponds to the subgroup called “section Livingstone” in **Table 3**.

Among the subset of 219 genomes, 214 belong to the subspecies *enterica* for the 58 serovars studied. The five genomes belonging to the other subspecies were used as outgroups for the cgMLST and pan-genome phylogenetic analyses. Both analyses revealed four groups in accordance with the results obtained with the first panel described above. Moreover, the comparison between the two phylogenetic approaches (cgMLST and pan-genome kmers) revealed the same composition of serovars and MLST profiles within each of the four groups of trees (Figure 3 and Supplementary Figure 3).

#### Polyphyletic serovars

Both cgMLST and pangenome kmers analyses revealed 25 polyphyletic serovars within the 58 serovars studied (Table 2). Of these 25 polyphyletic serovars, only the serovar Banana was scattered across four branches that distinguish four independent genomic lineages (three lineages in Group B and one in Group C). In our panel, we found seven serovars scattered across three lineages: Bareilly, Derby, Kottbus, Newport, Oranienburg, Reading and Saintpaul, with Reading shared between groups A and B, and Bareilly shared between groups A and C. Finally, the last 17 polyphyletic serovars were characterized by two lineages as shown in Table 2 and Figures 2, 3. Of these 17 serovars, 6 presented lineages in different phylogenetic groups: the serovars Bovismorbificans, Cerro, Infantis, Livingstone, Mississippi and Schwarzengrund. The serovars Bovismorbificans, Infantis, Livingstone and Schwarzengrund were shared between Groups A and B, Cerro was shared by groups B and C and Mississippi was shared by groups B and D.

**Table 2:** Polyphyletic and monophyletic serovars studied.

**Serovar and lineage L1 (MLST profile)**		**L2**	**L3**	**L4**	**Serovar and lineage L1 (MLST profile)**	
**Banana**	ST7024	ST4745	ST683, 5,220	ST1035	Brandenburg	ST20, 65, 249, 873, 2,577
**Bareilly**	ST5146	ST464, 1,612, 2,129, 2,270	ST203, 362, 909, 2,553		Coeln	ST1995, 2,015
**Derby**	ST682	ST39, 40	ST71, 72		Dublin	ST10, 73, 4,406
**Kottbus**	ST1669	ST582	ST212, 808		Enteritidis	ST11, 183
**Newport**	ST5, 118, 350	ST156, 166	ST31, 45, 46, 132, 157, 614		Gallinarum	ST78, 331
**Oranienburg**	ST23, 169, 174, 1,515, 1,675, 3,613	ST179, 1,392, 1,512	ST864		Goldcoast	ST358
**Reading**	ST1628	ST412	ST93		Hadar	ST33, 473
**Saintpaul** ^**a**^	ST95, 2118	ST49	ST27, 50, 680, 1,934, 3,602		Havana	ST578, 588, 872, 1,237, 1,524, 4,040, 7,676
**Bovismorbificans**	ST142, 377, 1,499	ST150			Heidelberg	ST15
**Bredeney**	ST214, 306, 897	ST505			Indiana	ST17, 2,040
**Cerro**	ST367, 1,593, 2,407	ST1291			Javiana	ST24, 371, 437, 589, 1,547, 1,674, 2,500
**Chester**	ST411, 1,954	ST343, 2,063			Johannesburg	ST471, 515
**Give**	ST516, 524	ST654			Kedougou	ST1543
**Infantis**	ST32, 603, 2,146, 2,283	ST79			London	ST155
**Kentucky**	ST152, 314, 2,132	ST198			Manhattan	ST18, 44, 2,200
**Livingstone**	ST543, 1,941, 2,247	ST457, 638			Minnesota	ST548
**Mbandaka**	ST3016	ST413, 1,602, 2,141			Montevideo	ST4, 81, 138, 195, 316, 699, 2,269
**Mississippi**	ST448, 5,834	ST425			Muenster	ST321, 2,692
**Muenchen**	ST83	ST82, 112, 1,606, 2,769			Napoli	ST474, 1,637, 2,019, 2,008, 2,095, 5,168
**Paratyphi B** ^**b**^	ST28	ST43, 86, 88, 110, 127, 149, 307			Ohio	ST329, 2,029
**Rissen**	ST2794	ST469			Panama	ST48
**Schwarzengrund**	ST2250	ST96, 322			Poona	ST308, 447, 964, 1,069, 2,889
**Senftenberg** ^**c**^	ST14, 210	ST185, 217			Stanley	ST29, 2,045
**Thompson**	ST26	ST2125, 2,417			Tennessee	ST319, 1,565
**Virchow**	ST16, 181, 303, 359	ST197, 1,750			Typhi	ST1, 2
Agona	ST13				Typhimurium^d^	ST19, 34, 36
Albany	ST292				Uganda	ST684
Anatum	ST64				Veneziana	ST2207
Braenderup	ST22, 311					

The polyphyletic and monophyletic serovars identified in the pan-genome phylogenetic analysis (kmer approach) are indicated with the corresponding MLST profiles. The polyphyletic serovars are indicated in bold. a: The difference between kmer and cgMLST phylogenetic approaches involves the Saintpaul MLST profile ST1934, which in the cgMLST tree is associated with ST49; b: the MLST profiles ST28, 43, 86, 88, 110, 127, 149 and 307 comprise the genomes of serovars Paratyphi B and Java; c: the MLST profiles ST14 and 185 comprise the genomes of serovars Senftenberg and S. 1,3,19:z27:-; d: the MLST profiles ST19 and ST34 comprise the genomes of serovar Typimurium and S. 4,[5],12:i:-.

The MLST profiles characterizing the different lineages of the polyphyletic serovars are compiled in the Table 2. The only difference between the cgMLST and pan-genome kmer analyses involved the genomes of serovar Saintpaul belonging to the MLST profile ST1934 that was observed in two different lineages (lineages II and III). All other data on the distribution of the MLST profiles among the different genomic lineages were concordant between the two types of phylogenetic analyses.

#### Description of genomic proximity between serovars

Nine subgroups of related serovars were observed in the cgMLST and pan-genome kmer analyses. In Group D, we found a subgroup — which we called “section Typhi” — comprising the genomes of serovar Typhi ST1 and 2, the genomes of the lineage Mississippi I (ST448, 5834), the genome of serovars Napoli and Veneziana (Table 3). Within Group A, we observed five subgroups, called sections “Livingstone,” “Enteritidis,” “Typhimurium,” “Newport” and “Muenchen.” The “section Livingstone” (section A1 in Figure 2) comprises the genomes of the lineage Livingstone I (ST543, 1941, 2247) and the genomes of the serovar Ohio. The “section Enteritidis” (section A5 in Figure 2), comprises the genomes of serovars Enteritidis (ST11, 183), Gallinarum (ST78, 331), Dublin (ST10, 73, 4406) and Berta (ST435). The “section Typhimurium” (section A4 in Figure 2), is composed of the serovars Typhimurium (ST19, 34, 36), Heidelberg (ST15), Coeln (ST1995, 2015), the genomes of the lineage Saintpaul I (ST49, 27, 50, 680, 3,602, 1,934) and the genomes of the lineage Reading I (ST1628). The “section Newport” (section A11 in Figure 2) is composed of serovar Kottbus ST212, 808, the genomes of the lineage Newport I (ST31, 45, 47, 132, 157, 614) and Newport II (ST156, 166). Finally, “section Muenchen”, (section A10 in Figure 2) composed of the genomes of the lineage Muenchen I (ST82, 112, 1,606, 2,769), Muenchen II (ST83) and the lineage Manhattan I (ST18, 44, 2,200). In Group B, there are two subgroups that we called sections “Montevideo” and “Bredeney.” The “section Montevideo” (section B9 in Figure 2) is composed of the genomes of the lineage Montevideo I (ST4, 195) and the genomes of the lineage Oranienburg I (ST179, 864, 1392, 1512). The “section Bredeney” (section B14 in Figure 2) is composed of the genomes of the lineage Bredeney I (ST241, 306, 505, 897), the genomes of the lineage Bredeney II (ST505), the genomes of the lineage Give II (ST654) and the genomes of the lineage Schwarzengrund II (ST96, 322) (Figures 2, 3). Within Group C, we observed the genomes of the lineages Banana I (ST7024), Bareilly I (ST5146) and Cerro I (ST367, 1,593, 2,407) with the genomes of the serovar Indiana.

**Table 3:** Identified subgroups.

**Genotype**	
**Section**	**MLST profile**
Livingstone A1	Livingstone ST543, 1,941, 2,247
	Ohio ST329, 2,029
Typhimurium A4	Saintpaul ST27, 49, 50, 680, 1,934, 3,602
	Coeln ST1995, 2,015
	Typhimurium ST19, 34, 36
	Reading ST1628
	Heidelberg ST15
Enteritidis A5	Enteritidis ST11, 183
	Gallinarum ST78, 331
	Dublin ST10, 73, 4,406
	Berta ST435
Muenchen A10	Muenchen ST82, 83, 112, 1,606, 2,769
	Manhattan ST18, 44, 2,200
Newport A11	Newport ST31,45,46,132, 157,614
	Kottbus ST212, 808
Montevideo B9	Oranienburg ST179, ST864, 1,392, 1,512
	Montevideo ST4, 81, 138, 195, 316, 2,269
Bredeney B14	Schwarzengrund ST96, 322
	Give ST654
	Bredeney ST241, 306, 897, 505
Indiana C	Banana ST7024
	Bareilly ST5146
	Indiana ST17, 2,040
	Cerro ST367, 1,593, 2,407
Typhi D	Typhi ST1, 2
	Mississippi ST448, 5,834
	Veneziana ST2207
	Napoli ST474, 1,637, 2,008, 2,019, 2,095, 5,168

The subgroups called “section Livingstone, Typhimurium, Enteritidis, Muenchen and Newport” belong to Group A. The subgroup referred to as “sections Montevideo or Bredeney” belong to Group B. The subgroup “section Indiana” belongs to Group C and “section Typhy” to Group D.

Interestingly, our analyses reveal that the polyphyletic serovars Banana, Bovismorbificans, Derby, Newport, Muenchen and Reading arose independently on divergent branches of the tree strongly associated with genomes of other serovars (Table 3). For example, the lineage Banana I (ST7024) arose in Group C and is associated with the genomes of the lineage Bareilly I (ST5146), the lineage Cerro I (ST367, 1,593, 2,407) and the genomes of serovar Indiana (ST17, 2040). The lineages Banana II (ST4745), III (ST1035) and IV (ST683, 5,220) arose in Group B. Nevertheless, the lineage Banana II is associated with the lineages Derby II (ST39, 40) and Livingstone II (ST457, 638). The lineage Banana III is associated with the serovar Tennessee (ST319, 1565) and the lineage Rissen II (ST469) and the lineage Banana IV is associated with the lineage Derby III (ST71, 72). On the other hand, contrary to the lineages Derby II and III, the lineage Derby I (ST682) arose in Group A and is associated with serovar London (ST155).

### Reference genome panel available in public databases

Given the selected set of high-quality complete genomes and the phylogenetic analyses carried out, we compiled a list of reference genomes with metadata and associated quality data (Supplementary Table 2). We selected 83 complete genomes from the initial set of 650 genomes, with 1 *S. enterica* subsp. *salamae*, 2 *S. enterica* subsp. *arizonae*, 1 *S. enterica* subsp. *diarizonae*, 2 *S. enterica* subsp. *houtenae* and 77 *S. enterica* subsp. enterica, representing 48 serovars and 71 MLST profiles.

## Discussion

SNP phylogenetic analysis is the most suitable approach to use in investigations of outbreaks with the goal of clustering epidemiologically related strains and calculating pairwise distance between genomes of the same serovar. However, when analyzing genomic diversity between different serovars, the cgMLST and the pan-genome kmer analyses are more appropriate approaches. The cgMLST predictions based on the 3,002 gene scheme are extremely stable (1). In a comparison of more than 100 000 *Salmonella* genomes, the cgMLST scheme can predict serovars with fewer errors than the slide agglutination reference method (1, 12). Moreover, the cgMLST method is more widely employed than SNP analysis with large genome panels because it is computationally less demanding (1). Owing to these advantages, the hierarchical clustering of cgMLST sequence types was also chosen in EnteroBase as the method of choice to map new bacterial strains to predefined population structures at multiple levels of resolution (9). On the other hand, accessory genes contribute to ecological specialization and the pattern of horizontal gene transfer among phylogroups can provide important complementary information (31), so that analysis of the pan-genome can lead a better picture of microbial organism proximity (32). Moreover, the pan-genome analysis of thousands of prokaryote samples is possible on a standard desktop without compromising the accuracy of results (33). Last, but not least, neither cgMLST nor kmer pan-genome analyses need a reference genome. This is a crucial point when analyzing the diversity of *Salmonella* genomes represented by a large number of different subspecies and serovars as in this study.

For this study, the 58 most frequently isolated serovars in France, EU and the US with their major sequence-type profiles were selected with a view to human health, animal health and the agri-food safety sector at the national and international levels. From the first set of 650 genomes analyzed for these 58 prevalent serovars, the final taxon sampling genomes was composed of five outgroups and 214 ingroup *S. enterica* subsp. *enterica* strains. The cgMLST and pan-genome kmer phylogenetic analyses both uncovered a deep split that delineates four sister groups within *S. enterica* subsp. *enterica*, including the two previously partially described groups (14, 25, 31). Although many of the relationships reconstructed in this study are consistent with previous reports, our taxon dataset provides a more thorough interpretation of polyphyletic serovars than any other study. The large selection of serovars and sequence-type profiles included allowed deeply appreciating the relationship between serovars, their genomic lineages and MLST profiles.

For each serovar, we included a larger selection of sequence-type profiles than previously. This large diversity gave a good overview of the complexity of the genetic diversity in *S. enterica* subsp. *enterica* and identified 25 polyphyletic serovars, 17 of which have never been described before, such as Banana, Bareilly, Kottbus and Reading for which we identified three distinct lineages, with the exception of Banana characterized by four lineages. Finally, among the 25 polyphyletic serovars identified, one serovar was characterized by four distinct lineage, seven by three and 17 by two distinct lineages. All of these serovars, such as Newport and Derby (13, 34), likely derive from multiple independent ancestors during the evolutionary history of *Salmonella*. Interestingly, *via* the whole-genome comparisons, we demonstrated for Derby (4, 13), as previously shown for Newport (35), that heterogeneity between lineages mostly occurs in the prophage regions and that lineage-specific characteristics are also present in the *Salmonella* pathogenicity islands and fimbrial operons. Further analyses are needed to investigate the other polyphyletic serovars identified in this study.

Although 25 polyphyletic serovars have been identified in our taxon dataset, there are probably more. For example, the serovars Agona, Havana and Montevideo were not identified as polyphyletic in our study, but have been described as such previously (14, 25, 36). The MLST profiles selection parameters applied in this study (i.e., for each serovar, we included the MLST profiles with 10 or more genomes in the EnteroBase database on 19 July 2021) did not make it possible to highlight the polyphyly of these three serovars. Furthermore, the number of distinct lineages for a polyphyletic serovar also depends on the taxon dataset selected. For example, we previously described four distinct lineages for the serovar Derby (37). In the dataset selected for the study of the diversity of the serovar Derby in France, the genomes belonging to ST39, even if most closely related to ST40 genomes (i.e., with an average of 3,962 SNPs and an SD of 20 SNPs), were identified as an independent lineage.

The influence of the dataset on the results was also observed on the genomic groups identified. In our panel, we underlined strongly supported subgroups that confirm previous observations (14, 25, 31, 34, 38). We called these subgroups “sections” to echo previous descriptions. For example, in this study, “section Typhimurium” comprises the genomes of *Salmonella* Typhimurium, Heidelberg and Saintpaul (14) and encompasses the genomes of *Salmonella* Typhimurium ST19, 34, 36, Heidelberg ST15, Saintpaul ST27, 49, 50, 680, 1,934, 3,602 as well as Coeln ST1995, 2,015 and Reading ST1628. When describing genomic serovar associations, the ST profiles must also be mentioned. For example, in the “section Typhimurium,” only the genomes of serovar Reading ST1628 belong to this section, because this serovar is polyphyletic and the two other Reading lineages ST412 and 93 are genetically distant.

This study confirms that, since the advent of WGS and advances in knowledge on the genomic diversity in *S. enterica* subsp. *enterica*, it is no longer possible to cite a serovar without referring at least to its ST group (1, 8, 38). The serovar-based nomenclature is still useful to maintain a link with data collected in the past and to continue to ensure smooth communication at the international level with testing laboratories and countries that have limited access to molecular techniques. Furthermore, European regulations regarding zoonoses stipulate that the serovar name is the mandatory reference nomenclature for *Salmonella*. Nevertheless, MLST profile information should be added to the serovar whenever possible. When the genome is available, the information regarding the MLST profile is easily accessible on open-access bioinformatics platforms such as EnteroBase or *via* the Center for Genomic Epidemiology (CGE) website (http://www.genomicepidemiology.org/) (39). For some laboratories that do not have the resources to perform WGS, it is still possible to determine the MLST profile with a PCR thermocycler and small first-generation sequencers. These labs can also send the PCR amplicons to sequencing companies for a lower cost than for a WGS system.

Furthermore, to avoid creating a two-tier system, it is the role of reference laboratories to provide genomic phylogenetic analyses, such as the analyses carried out in this study, to allow other laboratories to locate their strains on the trees.

Associating the historical name of the serovar with its MLST profile is an essential step toward the future of the nomenclature of *Salmonella*, which should have the advantage of clearly identifying the genomic lineage to which it belongs and indicating possible close links with other serovars (8, 9, 40). This association will also make it possible to obtain a more precise vision of the prevalence of certain serovars (and their genomic lineages) in various sectors, e.g., are all three Reading serovars prevalent in the poultry sector or is it only one of its three genomic lineages adapted to this sector?

In this study, we also provide a list of complete genomes that can be used as references and point out the absence of complete genomes for the following serovars: Banana, Kedougou, Mississippi and Veneziana. We also note the absence of complete genomes for the following lineages: Bareilly L2, Bovismorbificans L2, Chester L1, Infantis L2, ParatyphiB L1, Reading L2 and L3, Rissen L1, Saintpaul L2 and Schwarzengrund L1. We are currently sequencing short and long reads of these serovars to provide high-quality reference genomes for them.

Finally, in response to future outbreak situations or One Health surveillance of prevalent *Salmonella* and other emerging serovars, our study opens the way to a better understanding of the genomic diversity of *S. enterica* subsp. *enterica* and sheds light on the prevalent polyphyletic serovars at the national and international levels.

## Data availability statement

The datasets presented in this study can be found in online repositories. The names of the repository/repositories and accession number(s) can be found in the article/Supplementary material.

## Author contributions

Conceptualization and supervision: SC-S. Methodology, writing – original draft preparation, writing – review & editing, and visualization: EC and SC-S. Formal analysis: GI and EC. Data curation: EC, VN, and SC-S. All authors read and approved the final manuscript.

## Funding

This work was supported by the project Cross-sectoral framework for quality Assurance Resources for countries in the European Union (CARE), which has received funding from the European Union's Horizon 2020 Research and Innovation Programme under Grant Agreement No. 773830.

## Conflict of interest

The authors declare that the research was conducted in the absence of any commercial or financial relationships that could be construed as a potential conflict of interest.

## Publisher's note

All claims expressed in this article are solely those of the authors and do not necessarily represent those of their affiliated organizations, or those of the publisher, the editors and the reviewers. Any product that may be evaluated in this article, or claim that may be made by its manufacturer, is not guaranteed or endorsed by the publisher.

## Supplementary material

Supplementary Table 1 Accession number, taxonomic affiliation, MLST profiles and genomics data for the panel of 650 *Salmonella* strains analyzed. Genomes selected for the second 219 genome subset are shown in bold and gray fill color.

Supplementary Table 2 List of reference genomes with metadata and associated quality data for the *Salmonella* serovars identified as being the most frequently isolated in humans and the food sector over a period of 10 years (from 2006 to 2016) in the United States, Europe and France. Complete genomes selected for the second 219 genome subset are shown in bold and gray fill color.

Supplementary Figure 1 Venn diagrams illustrating the 47 *Salmonella* serovars identified to be common to the United States, Europe and France by logical relation analyses showed in Figure 1. The serovars are illustrated separately for agri-food isolates and human cases. To these 47 serovars, eleven others were added because they belong to the top 20 serovars from each country but were not common to all countries. The final list of 58 serovars retained for this study is showed in Table 1.

Supplementary Figure 2 Phylogenetic cgMLST distance tree of the 650 genome set of *Salmonella*. The tree is rooted on the *Salmonella entrerica* subsp. *arizonae, diarizonae, houtenae, indica* and *salamae* genomes. The tree is shown with branch lengths. Branches are shaded with different colors to distinguish the five groups identified.

Supplementary Figure 3 Phylogenetic cgMLST distance tree of the 219 genome subset of *Salmonella*. The tree is rooted on the *Salmonella entrerica* subsp. *arizonae, diarizonae, houtenae, indica* and *salamae* genomes. The tree is shown with branch lengths. Polyphyletic serovars are shaded in different colors. Complete genomes are highlighted with circles and contigs with triangles. Labels contain the serovar and the MLST profile of each strain.
