# Supplementary figures and images for: Polyphyly in widespread Salmonella enterica serovars and using genomic proximity to choose the best reference genome for bioinformatics analyses
Source: Front Public Health. 2022 Sep 8;10:963188. doi: 10.3389/fpubh.2022.963188 (PMC9493441; doi:10.3389/fpubh.2022.963188)

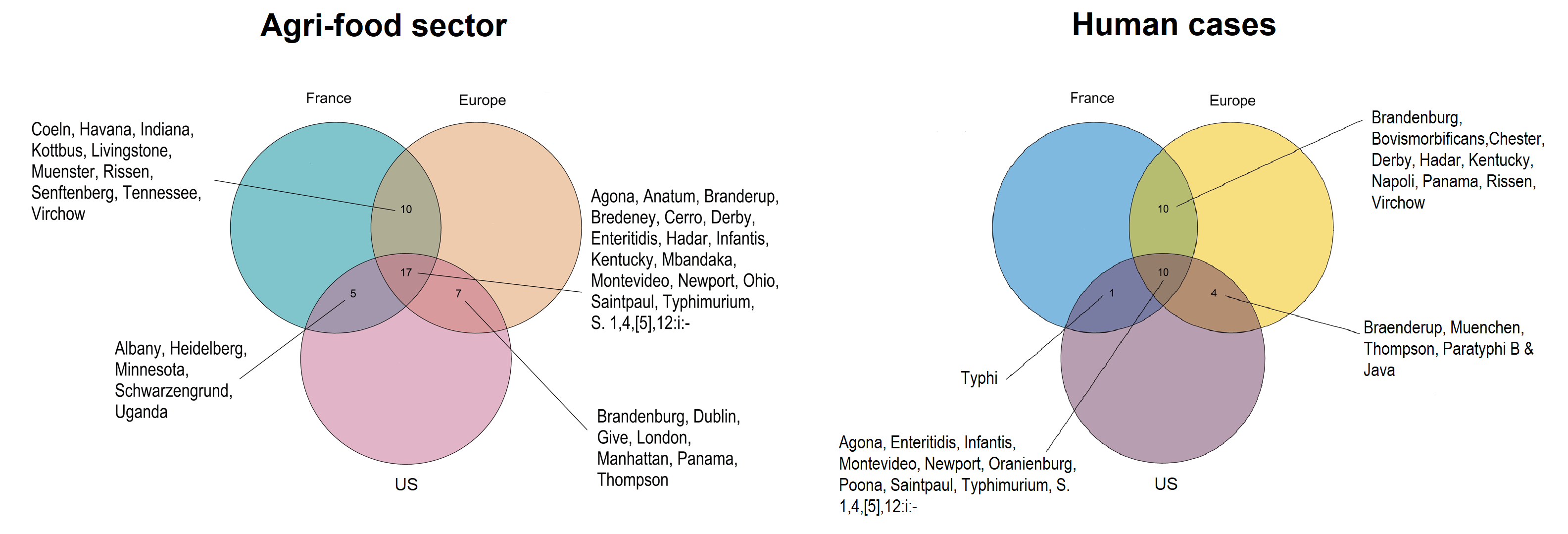

Supplement: Supplementary Figure 1 — Venn diagrams illustrating the 47 Salmonella serovars identified to be common to the United States, Europe and France by logical relation analyses showed in Figure 1. The serovars are illustrated separately for agri-food isolates and human cases. To these 47 serovars, eleven others were added because they belong to the top 20 serovars from each country but were not common to all countries. The final list of 58 serovars retained for this study is showed in Table 1. [file Image_1.tif]

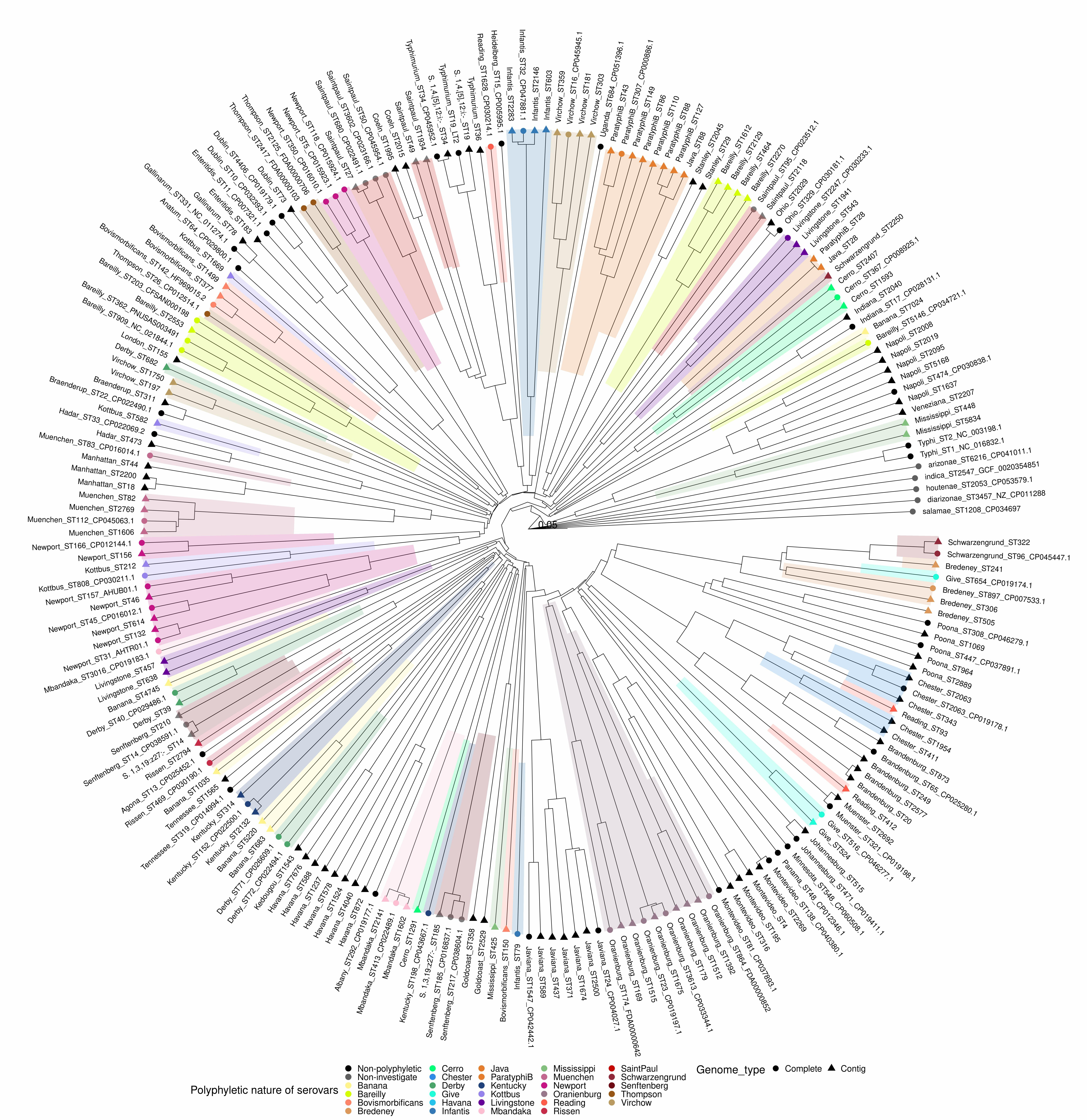

Supplement: Supplementary Figure 3 — Phylogenetic cgMLST distance tree of the 219 genome subset of Salmonella. The tree is rooted on the Salmonella entrerica subsp. arizonae, diarizonae, houtenae, indica and salamae genomes. The tree is shown with branch lengths. Polyphyletic serovars are shaded in different colors. Complete genomes are highlighted with circles and contigs with triangles. Labels contain the serovar and the MLST profile of each strain. [file Image_3.JPEG]
